# Medication refusal in residential aged care homes: A qualitative exploratory study

**DOI:** 10.1177/09697330261451370

**Published:** 2026-06-01

**Authors:** Stephanie M Garratt, Kathleen Tolton, Lyndal Bugeja, Alex Waddell, Elizabeth Manias

**Affiliations:** 1 School of Nursing and Midwifery, 2541 Monash University, Melbourne, VIC, Australia; 2 Living Experience Expert, Dementia Advocate, Brisbane, QLD, Australia; 3 Department of Forensic Medicine, 2541 Monash University, Melbourne, VIC, Australia; 4 Action Lab, Faculty of Information Technology, 2541 Monash University, Melbourne, VIC, Australia

**Keywords:** adherence, aged, ethics and dementia care, medication refusal, medication therapy management, nursing home

## Abstract

**Background:**

In Residential Aged Care Homes (RACHs), medication administration is typically carried out by aged care workers, rather than being under the control of older adults. Very little empirical qualitative evidence exists around situations where medication is refused by older adults.

**Research aim:**

To explore aged care workers’, older adults’, and family members’ perspectives and experiences of medication refusal in RACHs.

**Research design:**

A qualitative exploratory study design.

**Participants and research context:**

Between November 2024 and May 2025, participants were purposively recruited from two RACHs, and through a peak consumer group for people living with dementia and their families. Semi-structured interviews were conducted with aged care workers (n = 10), older adults living in aged care (n = 9), and family members of older adults (n = 7). Data were analysed using Braun and Clarke’s reflexive thematic analysis.

**Ethical considerations:**

Interview participation was voluntary, with either written or verbal consent obtained, depending on participants’ preferences. Identities were replaced with participant type and a numerical code. This study was approved by the Monash University Human Research Ethics Committee (MUHREC) (ID 41475).

**Findings:**

Three themes and four subthemes were developed regarding medication refusal in RACHs. These themes encompassed: (1) ‘Voices from different corners: medication refusals as more than just saying “no”’; (2) ‘the tightrope of role obligations – doing good, respecting choice, and avoiding harm’; and (3) ‘Competent to say yes, but not to say no: the erosion of the right to refuse’.

**Conclusions:**

Medication refusals in RACHs are clinically and ethically challenging for aged care workers, who use a variety of well-intentioned strategies to respond to these events. Given the complex nature of medication refusals, it is important that aged care workers take older adults’ decisional competence into consideration, balancing respect for autonomy with beneficence and best interests decision-making.

## Introduction

In Australia, residential aged care homes (RACHs), also known internationally as aged care facilities, or nursing homes, provide accommodation and nursing care for older adults who are unable to have their needs met in private homes.^1^ When an older adult moves into an RACH, administration of their medications typically becomes the responsibility of aged care workers, such as registered nurses.^2,3^ Medication administration is a routine clinical activity in RACHs, with each older adult having a particular medication regimen and specific instructions for how medications are to be administered.^4,5^ Older adults may take a passive or active role in their medication administration – they may make decisions about their medications, how they prefer to take them, or decide to refuse and not to take their medication at all.^3,6^ Whilst there has been an increased focus on supporting older adults’ autonomy and dignity in RACHs,^7^ less attention has been paid to the refusal of medication by older adults, and how aged care workers respond to these events. Medication refusal situations, particularly those involving an older adult with a dementia diagnosis, create an ethical dilemma for aged care workers, caught in conflict between respecting the older adults’ autonomy and dignity, or persisting with the medication to benevolently protect the older adult from potential medication-related harm.^8^ To date, empirical evidence around medication refusal in RACHs has been reported as an incidental or minor finding in quantitative studies focused on quality use of medications,^9–11^ or briefly in qualitative studies about medication adherence or covert administration.^12–16^ To better understand the tensions between respect for autonomy and beneficence during medication refusal events, and to build new knowledge around how these events are experienced and perceived, an in-depth qualitative exploration of medication refusal as a phenomenon in RACHs is required.

## Background

Definitions of medication refusal have primarily been developed for hospital contexts and perceptions of risk, with the term ‘refusal’ imbued with negative connotations, and the onus placed on the refuser to explain (if possible) why they do not wish to take their prescribed medication. Refusal of medication historically has been discussed as a form of dose omission, a medication administration error, and is described as a clinical concern.^11,17^ This emphasis as a clinical concern is because the dose has not been administered by the next dose time and a deviation from the prescription has occurred.^10,18^ Some investigators have attempted to distinguish medication refusals from negative connotations of error, arguing that they should be considered separately to omissions as they are initiated by the patient (or in this case, older adult), that individuals have the right to refuse (if they are informed of any risk and competent to make decisions about their medications), and an attempt was made to administer the medication.^19–21^ In comparison, medication errors are considered preventable in nature, and can lead to significant negative consequences for both older adults and aged care workers.^3,22^ Reasons for medication refusals can be multifaceted, and to consider them a form of error raises ethical concerns, particularly regarding respect for autonomy and best interests of older adults.^3,8,23^ Use of alternative terms such as declined medication or withdrawal of consent would place more emphasis on autonomy, but are not used widely, with medication refusal the dominant term internationally.

Regardless of whether medication refusal is defined as a form of omission/error, or not, refusals are known to be common in RACHs.^9,15,24^ Prior literature has described medication refusal as either active, if there is direct communication such as saying ‘no’ or pushing medication away; or passive, hiding medication, spitting it out, or avoiding the individual administering the medication.^8^ Importantly, older adults (if they are competent to make decisions about their medications and care) do have the right to refuse medication and are not necessarily required to provide a reason as to why they have chosen to do this.^8,15^ Commonly recorded reasons for refusal include not liking the taste of the medication, the size of the medication, cognitive impairment, fear of side effects, misunderstandings, or deciding that they no longer require the medication.^3,25^ Unfortunately, the recording of these reasons within medication documentation is known to be inconsistent, with between 52% and 99% of refusals lacking any notes about how/why they had occurred.^10,24^ It is important to include a reason for the refusal, as this can be used as an audit trail to support aged care workers’ responses and decision-making. Some reasons for refusal are able to be addressed if recorded, for example, changing a dose form if an older adult is refusing due to swallowing difficulties. Recording reasons for refusal supports shared decision-making between older adults and their wider care team during medication reviews.^24,26^

Medication refusals are a zone of ethical tension in RACHs. Aged care workers have a duty to protect older adults from harm and to support their best interests, but they are also have an obligation to support older adults’ autonomy and choice.^8^ Autonomy, decision-making without being influenced by others, is an inherent basic human right, but aged care workers play a fundamental role in facilitating, enabling, or limiting this right within RACHs.^27^ This duty creates a power imbalance between older adults (recipients of care) and aged care workers (providers of care). Some older adults may not be able to make autonomous decisions regarding their medication, they may be assessed or perceived as lacking competence due to cognitive impairment or a dementia diagnosis.^14^ However, it has been suggested that thresholds for competence should vary depending on the seriousness of the implications of a decision, that person-centric care directs us to empower older adults to have input into their care, given the heterogenous nature of this population group.^13,28^

Prior studies have indicated that aged care workers may persist with medication administration after it has been refused, regardless of the decision-making competence of the older adult in question, because they view refusal as carrying risk and not being in the best interests of older adults.^15,29–31^ The ideal for aged care workers is that older adults adhere to their prescribed medication, with those who do not adhere described in prior studies as requiring more nursing time, persistence, and problem-solving to avoid risk of adverse outcomes.^15,29^ However, this problem-solving is also known to lead to acts that minimise older adults’ autonomy and dignity, in particular, covert administration – the hiding of medication in food or beverages to ensure adherence.^12,32,33^ These acts raise significant ethical concerns regarding decision-making on behalf of older adults and respect for autonomy.

## Aim of the study

The aim of this study was to explore aged care workers’, older adults’, and family members’ perspectives and experiences of medication refusal in RACHs.

## Methods

### Design

A qualitative exploratory study design was performed as part of a larger multi-method program investigating the everyday experiences of medication administration in RACHs, and the strategies that could improve engagement and support older adults’ autonomy, dignity, and choice.

This study sought to understand the experiential process of medication refusal in RACHs, and was informed by a Deweyan pragmatist worldview, concerned with the interconnectedness of experience, knowing, and acting.^34,35^ Braun and Clarke’s reflexive thematic analysis was selected as the analysis method given its focus on researcher subjectivity and interpretive flexibility, which allowed for meaningful insights to be generated and a normative, dynamic consideration of reality – constantly shaped by human action and inquiry.^34,36^

The current study was performed and reported in accordance with Braun and Clarke’s (2024) Reflexive Thematic Analysis Reporting Guidelines (RTARG)^37^ and the Standards for Reporting Qualitative Research (SRQR).^38^

### Participants and research context

This study was conducted at two RACHs in Melbourne, Australia, both of which provided multiple levels of care for older adults, including secure dementia care. Both sites provided 24/7 nursing care for 60-80 older adults and primarily used Registered Nurses for medication administration. A convenience and purposive sampling was used to recruit a diverse sample of aged care workers, older adults, and family members across both sites.^39^ To increase representativeness and diversity of family members and older adults, the study was also advertised through a national consumer advocacy organisation that supports individuals living with dementia, their families, and carers.

Inclusion criteria were: aged care workers who regularly administered medication; older adults 65 years and over who had at least one medication administered regularly; and family members (anyone who identified as a relative or friend) of an older adult who had experienced medication administration in RACHs. Older adults with mild or moderate cognitive impairment (with/without a formal diagnosis) were eligible to participate. Exclusion criteria comprised older adults who experienced extensive agitation, anxiety, or pain; those with severe dementia, cognitive impairment, or mental illness;those who were receiving end of life care.

### Ethical considerations

This study was approved by the Monash University Human Research Ethics Committee (MUHREC), project ID 41475. Written or verbal informed consent was obtained before commencing the interview, with participants reminded of the voluntary nature of participation and their right to withdraw at any time. Participants were assured that their decision to take part had no bearing on their employment in the case of aged care workers, or care provided to them or their family member in the case of older adults and family members. Secure data storage and numerical codes were used to protect privacy and confidentiality.

### Data collection

Semi-structured interviews were conducted from November 2024 to April 2025. The interview guide was informed by prior literature around medication administration in RACHs and was developed by the lead researcher, a Health Sociologist, with input from another member of the research team, a Registered Nurse and Pharmacist with significant expertise in healthcare communication and medication safety. The lead researcher had a relationship with both RACHs, having conducted recent observational work at both sites. She conducted all interviews and made it clear to participants that although she had experience working with aged care workers and older adults, she was not clinically trained, and the intent of interviews was to hear about their views and experiences. However, with over a decade of experience, this enabled both an insider and outsider perspective, which also informed the overall research design and interview questions.

Semi-structured interviews were selected to explore the predetermined topic (medication refusals), whilst also allowing for exploration of spontaneous or unanticipated responses and thoughts from interviewees.^40^ Given the differing pre-existing power relations in RACHs,^41^ interview questions were tailored to be role-specific. A digital advertisement for the research was provided to the two RACHs to mail out, and an advertisement was displayed on the research webpage of a national consumer advocacy organisation. The research was also promoted by the clinical managers at both sites and the lead researcher at staff meetings, family member updates, and residents’ meetings.

Upon expressing interest, participants were provided with the study information sheet and consent form and were able to ask questions and seek clarification from the lead researcher or clinical manager at their site. Arrangements were made to conduct a one-to-one interview at a time and private location that best suited them, either in-person, or over the phone/Zoom videoconferencing. Participants were offered the option of providing written or verbal consent prior to the interview commencing. Verbal consent was offered to alleviate any concerns participants may have felt regarding signing a lengthy information sheet and consent form, and to ensure respect and dignity for participants with physical or cognitive challenges (e.g. hand tremors). Interviews and verbal consents were audio recorded. Interview transcription was done using a combination of Zoom technology’s auto-transcription, and manual transcription by the lead researcher. All names of individuals and RACHs were removed during the data cleaning process.

### Data analysis

Interview data was analysed using reflexive thematic analysis.^42^ Rigour and rich interpretation of meaning was achieved using a collaborative research process led by the lead researcher and involving the entire research team.^43^ The six phases for reflexive thematic analysis were followed. First, the lead researcher reviewed all transcripts, making notes in relation to the entire dataset. The research team then independently reviewed and made notes using one interview transcript, which had been conducted mid-way through data collection. Second, the research team independently generated short codes for the transcript, with each member using their particular lens – the team comprised a consumer advocate, registered nurse/pharmacist, implementation scientist, public health researcher, and health sociologist. The lead researcher then coded the entire dataset using NVivo (version 15),^44^ noting inductive descriptive and explanatory codes from each of the participants narratives. Third, codes were examined in order to develop initial themes, broader patterns of meaning, facilitated through the creation and refining of a thematic map. These themes were inductive, a combination of semantic (surface meanings of the data) and latent (actively interpreted by the lead researcher).^45^ Fourth, themes were collaboratively reviewed and revised by the lead researcher and senior researcher. Fifth, the scope and focus of each theme were appraised, and the wider research team provided critical dialogue to assist with the naming and refining of the themes. Finally, data extracts and the analytical narrative were written up, with themes now finalised.^42,43^

## Findings

In total, 26 individuals agreed to participate, including older adults living in RACHs (*n* = 9), family members of older adults (*n* = 7), and a variety of aged care workers (*n* = 10) ranging from Registered Nurses to Personal Care Workers. Participant demographics are presented in Table 1. Interview duration ranged from 9 min to 52 min, with a median of 23 min. All older adult interviews (*n* = 9) were conducted in person, in private rooms at their RACHs. Family members (*n* = 7) largely selected phone/Zoom interviews, with one choosing to respond to interview questions in writing.

**Table 1.** Participant demographics.

Role	Older Adults (Residents)	Family Members	Aged Care Workers (ACW)
Total interviewed, n	9	7	10
Gender, % female	89%	43%	100%
Length of interview, median (range) minutes	14 (9.55–52.17)	22 (15.13–35.20)	31 (21.1–41.32)
Length of stay in aged care, median (range) years	3.5 (1–7)	-	-

Aged care workers’ interviews (*n* = 10) were primarily conducted via Zoom or over the phone, with one in-person in a meeting room at their RACH. Aged care workers had spent a median of 10.5 years working in aged care (range 2–30 years) and had been in their current role for a median of 3.3 years (range 0.5–10 years). Seven aged care workers (70%) were qualified Registered Nurses. Other roles included Enrolled Nurse (*n* = 2, 20%) and Personal Care Worker (Unlicenced aged care worker, certified to administer medications) (*n* = 1, 10%).

Analysis identified three main themes, and four subthemes (Table 2). Themes and subthemes are supported by key verbatim quotes from participants, identified using their role and an assigned number (e.g. ACW 1).

**Table 2.** Overview of themes and subthemes.

Themes	Subthemes
(1) Voices from different corners: Medication refusals as more than just saying ‘no’	​
(2) the tightrope of role obligations – doing good, respecting choice, and avoiding harm	• Upholding the right to refuse: Putting older adults in the driver’s seat
	• Navigating cognition and competence
(3) competent to say yes, but not to say no: the erosion of the right to refuse	• Softly softly: Using knowledge to question and inform
	• Craving certainty: Deciding for older adults

### Theme 1. Voices from different corners: medication refusals as more than just saying ‘no’

The majority of participants were aware that older adults could refuse medication in RACHs, but had divergent perspectives on the frequency of these events, who refused medication, and why. For aged care workers, their perspective was uniform – refusals were a challenge they experienced ‘on a daily basis, but not necessarily the same resident’ (ACW3). Their accounts underscored not only the strain experienced through catering for a wide variety of older adults and care needs, but also the importance of older adults understanding what their medications were for. For example, the most common medication types mentioned were laxatives and diuretics, where aged care workers appreciated when older adults reported not needing the medication, and gave a clear reason for refusal.

**“**…they might be having an off day, or they just don't want to take maybe a particular tablet for a particular reason. […] I used to have a gentleman that didn't want to take his diuretic sometimes when he was going out because, for obvious reasons, he didn't want to be going to the toilet all the time.”—ACW7

However, aged care workers were also quick to note that not all refusal situations involved clear communication or a reason from older adults. These situations were more likely to be complex and required additional work and time from aged care workers. This was not because a reason was necessarily required, but because aged care workers felt the need to ensure that older adults did mean to refuse and they understood the situation. This was compared to a refusal being the result of an external cause, such as the use of an unfamiliar aged care worker:

“ […] very randomly, they'll do it, they might do it because, for example, here in [dementia care] there was a lady who didn't want to take medication for me, and probably it was to do with the fact that I was a very new face.”—ACW2

In contrast, older adults presented a more complex blend of accounts around medication refusal in RACHs. Older adults who participated in this study either framed themselves as the expert on their medications (able and supported to make their own informed choices and give reasons for a refusal), placed their trust in the aged care workers and medical professionals and did not refuse medication, and were surprised that ‘do people do that? I’d be amazed to think why’ (Resident 3).

“I've refused it when we had agency [temporary] staff and they didn't know what they were doing, and because I've still got what wits about me. Luckily, I could say I don't have that when they tried to give me something [medication] that wasn't mine…” –Resident 1

Older adults who refused regularly were framed by their peers as less competent ‘others’, who could be unreasonable, and did not understand their medication. Whilst some older adults described situations where ‘you’re just sort of listening to them saying, I don’t want to take that tablet’ (Resident 4), the majority of examples given involved physical forms of refusal.

“Well, I wasn't in the room, but I walked by and I've heard the staff say, you need to take this one and that [the resident said] I don't want any, you know, the hands fly out everywhere. And the nurse will take the medication [away]. You can't afford to leave it sitting there. Because you know they're not going to take it.”—Resident 9

**“**A lot just…throw it [medication]. A lot. I see them, they throw one over their shoulder […].”—Resident 6

Rather than focussing on medication refusals themselves, family members described their loved ones living in RACHs as either highly opinionated, or highly adherent when it came to their prescribed medication. The most common response from family members when asked about their loved ones ever refusing medication was that they would ‘never say no to medication’ (Family member 3). High trust was placed in the aged care workers caring for their loved ones, and family members expected that medications were explained and managed correctly.

“I don't pretend to understand how the medication works, or anything like that. But what I do think is important is understanding that the medication being taken, the medication is being prescribed is for a valid reason, and that it is being administered and supervised correctly. That's the important part to me”. —Family member 1

### Theme 2. The tightrope of role obligations – doing good, avoiding harm, whilst respecting choice

Older adults and their family members placed importance on aged care workers treating older adults as individuals, trusting that they would receive quality care, respect, and good communication about medications.

“I'm not backward in coming forward. So, if there's something I need to say, I say it, you know. And that's fine. I've got a good relationship with the nursing staff and the doctor.”—Resident 1

Participants emphasised that older adults should be treated as individuals; that they should be supported to make decisions, and that doing good was a core aspect of aged care workers’ role.

“I think my family, my grandmother, especially in this, are in this very lucky position where, the staff members at the aged care facility [RACH] haven’t done anything wrong, like made any mistakes. And they're really kind and caring […] I'm just happy that there's, like, effective communication for both sides [older adult and family].”—Family member 3

“Basically, we're giving the best possible care we can. Holy, yeah, for the residents, everything that we do is for them. It's their home. […] It's the last part of their life, whether it be long or short. So, yeah, it's just about caring for them in every possible way, at the highest standard that we can and making them the happiest that they can be, or the rest of their time that they're going to spend here”—ACW7

Aged care workers were aware of their duty to provide good quality care and minimise risk of medication-related harm to older adults, which influenced their initial responses to medication refusal. Safety was a key concern, with several aged care workers referring to the need to ensure medication was taken, and to conduct risk assessments if a refusal occurred.

“Some residents, they will give you a reason most of the time. But if we have some residents, you know, they don't take all medication. They [doctors] chart it, they still don't take it. So, for that situation, you do a risk assessment, which is a lot of…let them know what the risk is if you don't take your regular medications, yeah. And if you're happy to know this risk and you still want to take this risk, so just have the assessment document there and let the family know. Let the doctor know that if they refused. That is a choice.”—ACW6

“[…] if they choose not to take it […] since if this is their choosing, then most likely it would cause harm if they don't take this medication when it's supposed to help them instead. Yeah, so missing it will actually cause further harm. You know, like antibiotics, like these are important.”—ACW9

### Upholding the right to refuse: Putting older adults in the driver’s seat

The right to refuse medication was frequently discussed by participants around supporting older adults’ choice, dignity, and respecting autonomy. Older adults trusted that when they refused medication, that their wishes would be respected, that it was their right to say no and have input into what they received.

“I'm quite aware that I can say no [to medication] if I don't want it.”—Resident 3

Aged care workers described a shift in attitude towards the right to refuse, that previously refusals had been viewed as worthy of an incident report. Refusals were still associated with risk, but equally seen as ‘it’s very much their choice if they don’t want to take their medications’ (ACW2), and that ‘we can’t force them’ (ACW10).

“I think it's good, like, if we respect their choice […] Maybe sometimes they don't want to take something. And if we say, no, you have to take it […] we can just explain the benefits and everything, but…and then we have to respect their choice. You know, if we won't be doing that, I think it will cause like, you know, agitation to them, which is obvious, and I think it's good, we should focus more on the personalized person-centred care, rather than like applying the same rule to everyone, it's not possible”—ACW4

### Navigating cognition and competence

It was common for aged care workers and older adults to emphasise older adults’ ‘cognition’ or ‘mental status’ as the key consideration when deciding whether to uphold a medication refusal or not, rather than using that term ‘competence’. Upholding a refusal from an older adult who was ‘cognitively fine’ (ACW9) was still a concern if the medication was important, but less so compared to a refusal from an older adult with a dementia diagnosis who might not understand what they were refusing.

“If you work in, like dementia or like with residents with acquired brain injury, I think, or even psychosocial […] may be suspicious or paranoid about the medication [or] with sundowning [refusal of medication] would have a bit to do with, like behaviours […].”—ACW5

You know, it's that, you know, what I witness with Mom is she has the thought in the mind, she knows what she wants to say, but she can't get it to her lips and that's a total disconnect. So, I think sometimes what comes out isn't necessarily what they wish. Whereas with other patients, I would say absolutely, you know, if you're of sound mind, you know and have the ability to make those decisions, absolutely, and I'd almost go as far to say that at a place like [aged care site], I would expect that they would probably do that [respect the decision to not take medication].”—Family 2

Older adults expressed pity and concern for those with a dementia diagnosis, as these individuals might not always know what medications they were taking, and why it was important to take them. However, they were also aware that even with a dementia diagnosis, there could still be a reason behind why they were refusing medication.

“[…] they should know what they're taking. What's going in their mouth. And half of them don't know. They just take them. Because of dementia. They don't know. They wouldn't ask what they're for or anything [medication]. I feel sorry for them. Because they could be given anything or decide not to take it.”—Resident 8

“I mean, those with dementia, they do need it [medication] when they don't know what they're saying or what they're doing. And making a hell of a lot of noise. So they do need that quietening down period. And they don't want to be a zombie. No. And they've got that much [awareness] that they know they don't want it [medication].”—Resident 9

### Theme 3. Competent to say yes, but not to say no: the erosion of the right to refuse

Although participants were cognisant of the right to refuse medication, a spectrum of response strategies were described by aged care workers, aimed at promoting informed consent, convincing, or overriding an older adults’ refusal of medication. These strategies are presented in Figure 1. All strategies adopted by aged care workers were born of a desire to adhere to an older adults’ prescription, to act in their best interests, and to protect them from potential harm (a risk of medication non-adherence). Older adults that were interviewed did not discuss how aged care workers responded to medication refusal – their experiences limited to either not refusing medication, or if they did, aged care workers had respected their decision and did not pursue any additional actions. Importantly, although family member participants were largely not aware of their loved ones refusing medication, they assumed they would be contacted or made aware if the situation arose.

“It's just you have to listen and you have to pick, and put a little bit of creative thinking that…how can you manage this situation [the refusal]?”—ACW1

“[…] the communication side of letting people know what they let us know about, I don't think takes a lot of time. It's about being aware of it and making a little bit of effort.”—Family 1

**Figure 1.**
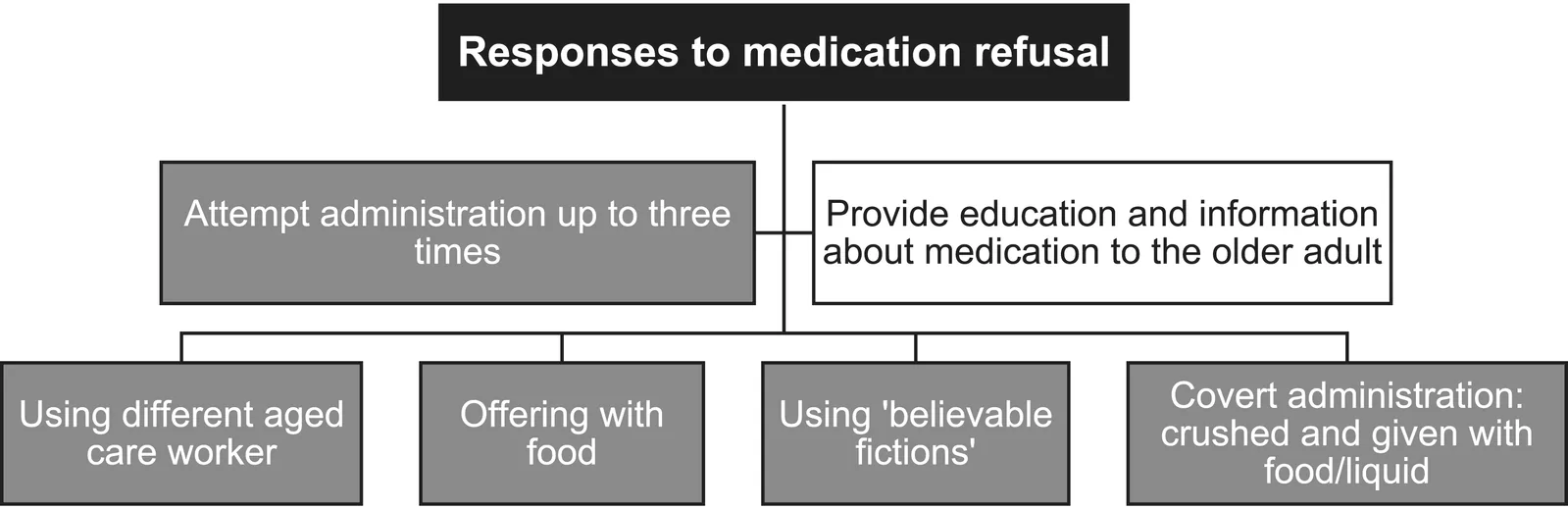
Responses to medication refusal in aged care facilities.

### Softly softly: Using knowledge to question and inform

The first line of response to a medication refusal involved aged care workers providing information to older adults about the medications in question, and the reasons why they were necessary. This provision of information was tailored to the older adult and assumptions about their competence and understanding. The intent was not just to ensure an informed decision was made, but to ideally change the older adult’s mind.

“So if someone refuses, then you know they have to find out why the resident is refusing. That's the first thing that they need to do. And then you know if, if try to educate, if the reason why they're not taking is, like, you know, lack of knowledge.”—ACW3

“[…] depending on their cognition, if they've got a bit of dementia, and you can keep it basic and just tell them what it's for and that they'll feel better once they've had it generally, but if it's someone that's a bit more cognitive [possibly considered competent], then, yes, we start to get into why don't you want to, you know, what's going on today? How come you don’t want to take this?”—ACW7

### Craving certainty: Deciding for older adults

Aged care workers were quick to emphasise that they could not force older adults to take medication, but all discussed strategies they used to work around a medication refusal. Many of these strategies sat within an ethical grey area, where respect for autonomy was at risk of being minimised in order to meet older adults’ perceived best interests (as perceived by aged care worker). It was unclear if these strategies were solely used on those living with a dementia diagnosis or cognitive impairment, or if they were applied to all older adults irrespective of their decisional competence.

“[…] you utilize a lot of strategies. You could explain first, if they don't understand, you try to redirect their attention, especially if they've got dementia [...] Or you can find things that make might like, make it more appealing to them, like food. You mix it with food, see if they would tolerate it better. Or if we could, you know, if, if, if they're aware that you're giving medications and they would refuse sometimes, you know, it's really looking for strategies, especially if that medication is really necessary for them.”—ACW9

Other than offering information and asking questions to try and ensure an informed decision was made by older adults, the most common strategy discussed regarding medication refusal was to keep offering the medication, at least or up to three times. The reasoning behind this was to give the older adult time and space to change their mind, or because they might forget they initially refused. On the second attempt, food might be offered, another aged care worker might be used, or if the older adult continued to refuse, certain medications might be negotiated over others.

“I will still attempt at least three times, without getting in their face, of course. But like, I'll try a different approach…so say, if they've refused it before breakfast, [..] I'll take the breakfast in the room, and maybe then I'll say, you know, while we're taking this, you know, maybe, will they take this? Or maybe I'll try to say, Oh, this is really important. You know […] maybe we'll just take the small tablet. Or maybe what I try to negotiate is things like, maybe anticoagulants or antihypertensives, which are really, like, I wouldn't worry so much about, things like, maybe, I don't know, Panadol. I'll try to prioritize. And I'll say, maybe, how about if I take just if you want to take just the small tablet […]”—ACW2

Aged care workers also described crafting small, believable fictions that would prompt an older adult who may lack decision-making competence to revise their refusal and accept their medication.

“[...] if we say [family member] has spent money on the medication you should take it because [they’re] paying for it and you shouldn't be wasting it...” –ACW1

Covert administration was commonly described, involving crushing medication and administering it with yoghurt or other mediums without older adults knowing. Some aged care workers emphasised there should be family and/or prescriber approval for this process, although it was not clear if this approval was gained long-term and noted on the older adult’s medication chart, or if approval was sought each time they refused.

“[…] if the resident [living with dementia] says, no, I don't want to take my meds, and the family is aware and it's been happening, then usually family or doctor, they say that mix it with food or mix it in a juice and give it to the resident, because it makes a difference when they have it or not have it, that they don't know what they are actually doing. And then there is protocol of mixing the medication in something, but then we should have the consent from family or doctor saying that.”—ACW4

“[…] in that moment you know to mix with the tea and juice, anything they like. If you are going to do it, it has to be crushed into something that will definitely enjoy.”—ACW6

## Discussion

The aim of this study was to explore older adults, family members of older adults, and aged care workers experiences and perspectives of medication refusal in RACHs. Previously, medication refusals have been noted in quantitative and qualitative studies as a cursory, minor, or incidental finding, within examinations of medication dose omissions in age care facilities and hospital settings,^9,10^ or modification of medication dose forms (including covert administration of medication) in RACHs.^3,12,15^ Medication refusal is more complex than simply saying ‘no’ to medication. Our study shows that older adults and family members hold divergent views on who should be supported if they refuse medications, and why. Also, whilst all participants placed value on treating older adults as individuals, in line with person-centred care, tensions arose regarding older adults’ decisional competence due to aged care workers’ obligation to protect older adults from harm. This study also confirms, and supplements existing knowledge about actions in response to medication refusal, such as covert administration of medication, and the embedded tension in RACHs between respect for older adults’ autonomy, and their best interests (as perceived by aged care workers) regarding medication use.

The varying narratives around why medication refusals occurred, the frequency of these occurrences, and who refused medication underscore the challenge associated with providing clinical care to a highly diverse population of older adults, some of whom may or may not understand the medication they are taking, or may be unable to provide a reason as to why they wish to refuse medication. Participants in our study demonstrated a belief that refusing prescribed medication, especially physically refused (e.g. saying no, or throwing the medication) without a clear rationale, was inappropriate or morally undesirable, and that what was prescribed should be adhered to insofar as possible. Yet it may be that older adults are unable to articulate a perfectly sound rationale for refusal due to a chronic condition or cognitive impairment – it is still important that aged care workers consider why they may be refusing medication. Family members also acknowledged that medications were prescribed ‘for a reason’, and aged care workers felt driven to ensure that medication was taken in accordance with the prescription. Medication prescriptions not only operate for therapeutic purposes, but are a form of tangible social contract between prescribers, aged care workers, and older adults living in RACHs. Prescriptions in these settings represent professional authority and hold significant symbolic value,^46^ with aged care workers in a position of power as the administrators of medication. The high trust placed in prescriptions and aged care workers may negatively influence older adults to passively agree to medications or decisions that are not in line with their preferences, limiting relational autonomy, confidence, and communication.^47,48^

Historically, the more passive, unquestioning term ‘compliance’ was used regarding medication-taking behaviours, rather than the more active ‘adherence’.^49^ Even if aged care workers desire to support older adults’ autonomy and involvement in decision-making regarding medications, they still remain in strict control of prescriptions and the medication administration process.^48^ Older adults that could provide an active, clear rationale for their refusal of medication felt supported and respected by aged care workers. In these cases, a medication refusal, well-informed and active in nature, can lead to a dose being refused or altered to suit personal needs,^50^ in order to exert control over a treatment and its effects on the body or daily life.^16,51^ This situation is particularly true of laxatives and diuretics, where an older adult may feel sufficiently compelled and confident to refuse a dose, despite the prescription, because the means (a non-interrupted social outing) justify the ends (being non-adherent once, or multiple times). Ultimately, medication adherence remains a significant concern in RACHs, given that older adults are typically prescribed multiple medications, may be living with multimorbidity, and have highly diverse care needs.^52^ Older adults may not be able to clearly articulate the reasons behind a refusal due to cognitive concerns, adverse effects of medications, or chronic conditions. It is important that those who refuse medication with or without a clear rationale are supported to have input into their care, insofar as they are able, balanced against aged care workers’ professional motivations to administer what has been prescribed.

As a theme, ‘the tightrope of role obligations’ exposes the inherent conflicts, potential cognitive dissonance, and uncertainty surrounding medication refusal and the provision of person-centred care in RACHs. Person-centred care (placing the individual care recipient at the centre of care decisions, an equal partner in their care) has been widely touted as the gold standard for RACHs internationally.^53^ All participant groups were clear that older adults should be treated as individuals, with their choices and rights respected. Yet contravening this, aged care workers were still concerned about refusal of medication irrespective of an older adults’ competence. Decisional competence, the ability of older adults to understand, appreciate, and reason to make a rational informed choice,^28^ was not discussed well by participants in the context of medication giving and refusal. Examples of refusal were provided where decisional competence was either presumed/wholly present, or lacking entirely. Decisional competence is known to sit on a sliding scale, dependent on the state of the older adult at the time and the cognitive complexity of the situation, and is not fixed.^54,55^ However, institutional constraints, safety concerns, and conventional practices, such as the rules and strict control of medication giving processes, limit aged care workers’ ability to deliver nuanced individualised care that takes decisional competence into full account.^53^ As a result, and in the absence of a family member or prescriber being readily available, whilst under time pressures to complete medication-related tasks, determination of decisional competence and older adults’ best interests in RACHs is largely the purview of aged care workers. Their potentially reactive decision-making and risk-averse responses can either support older adults’ autonomy, or bring about benevolent paternalism, as they find themselves thrust into the role of surrogate decision-maker, balancing risks and benefits through the lens of their own concept of the good.^28,56–58^ This raises ethical concerns, if aged care workers are under time constraints and workforce pressures, and are, as found by the current study, concerned about the possible consequences of non-adherence irrespective of an older adults’ decisional competence. There is risk that, without clear organisational guidance, concerns about competence become a slippery slope,^31^ where older adults who are competent to consent to, or refuse medication may be denied input into their care.^33,59^

Despite heavy emphasis on person-centred care, RACHs are structured around routines of daily life and normative medical decision-making, creating an environment where opportunities to make decisions and assess competence as ongoing processes are limited.^27,48^ More active and nuanced consideration of older adults’ decisional competence, and how to practically implement shared or supported decision-making around medication use, is needed from aged care workers in order to better support older adults’ decision-making insofar as they are able, given the structured environment. Despite emphasis that aged care workers were unable to force older adults to take medication, noting the right to refuse, this study has found that aged care workers may undertake a range of well-intentioned, but potentially coercive and paternalistic strategies in response to medication refusal. It has been established that older adults may struggle to maintain their autonomy in healthcare settings when well-meaning others seek to exercise paternalism to achieve a particular outcome – in this case, medication adherence.^48^ RACHs, as institutions, are designed to cater for older adults that may have reduced or fluctuating competence, but organisational and clinical processes leave little room for aged care workers to create space to attend to older adults’ remaining decisional competence.^48^ Aged care workers described strategies to promote medication adherence including trying to administer medication multiple times, use of believable fictions (therapeutic lies), and covert administration, in order to further older adults’ perceived best interests. In particular, covert administration of medication was commonly mentioned as a strategy to respond to older adults’ refusal of medication, especially for older adults living with a dementia diagnosis or cognitive impairment. This ethically and clinically fraught practice has been documented over the past 20 years in RACHs, and involves crushing and administering medication in food or liquids.^3,59^ Aged care workers noted that covert administration should be authorised by an older adults’ family member/proxy, and their prescriber, but prior studies note that covert administration can also occurs as an unauthorised practice, with aged care workers making the decision in isolation.^12,60^

Concerningly, given that covert administration is considered to be less time-consuming and challenging for aged care workers compared to coercion (e.g. trying three times) in response to medication refusal, RACHs are typically left to create their own policies and procedures around this practice.^12,15,25^ There is no clear guidance internationally on covert administration of medication in particular, but prior studies report that this practice is carried out by aged care workers who may have limited knowledge or training around how/which medications can be safely crushed.^3^ It was not clear if the strategies described by aged care workers were used just for older adults with a dementia diagnosis and wholly lacked decisional competence, those who refused medication in general, or if authorisation was obtained from family or a prescriber each time or as an ongoing permission.^56,61^ Overall, RACHs, and the older adults who reside in these sites, have evolved significantly, with older adults entering care with a wide range of capabilities and expectations. This creates a significant challenge for aged care workers, tasked with protecting older adults from harm, promoting their best interests, providing high quality clinical care, whilst also charged with respecting older adults’ autonomy. The need for essential medication may legitimise impinging on older adults’ autonomy if they refuse medication, but lack of competence to consent should be a key condition for use of coercive or paternalistic methods.^58^ The use of ethically fraught strategies that promote medication adherence, although well-intentioned, risk eroding older adults’ trust, and undermine their autonomy and dignity.

## Strengths and limitations

A strength of this study is its varied sample, with aged care workers involved with medication administration from all role levels, and older adults and their family members making up over half of the sample. Their diverse experiences and perspectives add depth to this study. The two RACHs comprising the study were located in metropolitan areas. Their values and policies around medication administration and refusal may differ from other RACHs, particularly in regional and remote areas, possibly limiting transferability. When interviewed, in line with their experiences, aged care workers provided significantly more detail around responses to medication refusal, with older adults and family member unable to provide specific examples or insights. As a result, the aged care worker voice is dominant, particularly regarding theme 3.

## Conclusions

This qualitative study identified that medication refusal in RACHs is complex, especially as older adults may or may not provide a clear reason for their decision to refuse medication, and their decisional competence may not be well understood by aged care workers. Medication giving and refusals create tensions and challenges which aged care workers must navigate, whilst also striving to respect older adults’ autonomy and providing safe, good quality care. How aged care workers respond to medication refusals can either support or undermine older adults’ autonomy and right to refuse. There is a need for ethically informed guidance and education around older adults’ decisional competence and how far the right to refuse medication extends in RACHs.

## Data Availability

The data that support the findings of this study are available in a de-identified format from the corresponding author upon reasonable request, and subject to appropriate ethical approvals.*
